# Gas Furnaces vs. Coal or Coke

**Published:** 1886-03

**Authors:** H. C. Land


ARTICLE VII.

### GAS FURNACES vs. COAL OR COKE.

BY H. C. LAND.

That the gas furnace for dental operations is destined to supercede the coal or coke there can be no doubt. To get rid of the disagreeable features, such as the handling of coal or coke, the dirty ashes, etc., add to this the much greater loss of time in accomplishing the same results as well as the uncertainty of success that may arise for want of a better draft, poor coal or coke, and the unfavorable condition of the weather. To say that the gas furnace is absolutely free from all the above objections, is a valuable count in its favor, and to add that it will produce superior results in less time with unerring precision is all that could be desired. Heretofore the serious trouble with gas furnaces has been the liability to injure the color of the body and enamel. After a long and careful series of experiments, I have at last discovered the secret of the trouble, and in a very simple and pratical way have entirely overcome them. Therefore it is with much pleasure I now deem it my privilege to announce to the profession the technical reasons. In the use of the various hydrccarbons which are at our command olefiant gas $C_2 H_4$, gasoline $C_2 H_4$, are the most available, from which we must secure the necessary degree of heat. The philosophy of the combustion of these two substances may be illustrated in the following manner: Draw three parallel lines, an inch apart, one above the other, this will represent the interior of the furnace, estimate the burners as being set one inch beneath. The first line will represent the bottom of the muffle, the next line one inch higher on the outside of the muffle, and the third line the top of the muffle. We have here 1st, 2nd, and 3d stages of combustion. In the inch below the muffle one atom of oxygen is taken up, producing di-oxide of carbon; in the next stage two atoms of oxygen unite, producing mon-oxido of carbon, and in the third and last stage, three atoms of oxygen are taken up producing carbonic acid, this is perfect combustion. Now, if we take into consideration the fact that in order to secure 2,800 degrees of heat it is necessary to have a bellows that will supply a constant blast of one pound pressure to the square inch, it becomes evident that this force is capable of driving the di-oxide of carbon completely through the pores of the muffle, especially when it is driven directly against the bottom, this product enters the body and remains in it, and as it takes about 2,800 degree to eliminate di-oxide of carbon—the biscuiting being completed at a much lower degree—therefore, when the case is subjected to a degree of heat sufficient for enameling, this di-oxide or it may be mon-oxide of carbon, comes out in the shape of numerous small bubbles, this is what we call gasing the teeth. To overcome this difficulty my first step was to force a current of heated air into the muffle and in this manner drive the di-oxide of carbon out. This was successful in a degree, but I soon discovered that too much air from the excess of oxygen would also bleach the enamel. I then secured an atmosphere of pure nitrogen and injected this into the muffle, it being a neutral gas, this was eminently successful and demonstrated the fact that porcelain baked in an atmosphere of pure nitrogen is absolutely perfect. The next step was to secure the nitrogen in some simple way, this has been accomplished and gives no additional cost or trouble to the furnace. To those who wish to see practical demonstrations they are welcome to my laboratory at Detroit, or in due time shall be able to give practical demonstrations before the dental associations. The advantage to be able to step into one's laboratory and within the space of from fifteen to twenty-minutes bake a set of porcelain teeth, or it may be to bake a porcelain crown fast to a platinum tube that is intended to cap over a root, cannot be over-estimated. A lady called at my office with her seventeen-year-old daughter, both the superior lateral incisors were not more than one-third their proper size, and in contrast with the other well developed teeth were a disfigurement. To remedy this trouble, two platinum tubes were fitted over the teeth; the next step was to take two ordinary plate teeth and grind them to mere shells, this secured the size and color, then they were baked to the tubes by using a little fresh tooth body, the result was a complete success, the joint at the margin of the gum could not be seen. The subject of this circumstance tells her schoolmates that her teeth have overcoats on.

The next experiment will be some porcelain and platinum bridgework by stamping the cusps of molars and bicuspids, then fill them with body and bake, then solder them on to the bridge work, After this is done take the ordinary plate teeth, grind the crowns down so that they are level, then fit them to the bridge-work, the whole can then be placed in the furnace and by previously filling all the interstices between the teeth and the platinum crowns a most beautiful piece of bridge-work can be secured. My object in presenting these cases is to awaken the profession to the fact of the dawn of a new and brilliant future for dental prosthesis.—*Dental Register.*

---

628 W. Lake St., CHICAGO, ILL., March 3d, 1886.

Editor of AMERICAN JOURNAL OF DENTAL SCIENCE:

